# Investigation of 2,4-Dihydroxylaryl-Substituted Heterocycles as Inhibitors of the Growth and Development of Biotrophic Fungal Pathogens Associated with the Most Common Cereal Diseases

**DOI:** 10.3390/ijms25158262

**Published:** 2024-07-29

**Authors:** Klaudia Rząd, Aleksandra Nucia, Weronika Grzelak, Joanna Matysiak, Krzysztof Kowalczyk, Sylwia Okoń, Arkadiusz Matwijczuk

**Affiliations:** 1 Department of Biophysics, Faculty of Environmental Biology, University of Life Sciences in Lublin, Akademicka 13, 20-950 Lublin, Poland; klaudia.rzad@up.lublin.pl; 2 Institute of Plant Genetics, Breeding and Biotechnology, University of Life Sciences in Lublin, Akademicka 15, 20-950 Lublin, Poland; aleksandra.nucia@up.lublin.pl (A.N.); grzelak.wer@gmail.com (W.G.); krzysztof.kowalczyk@up.lublin.pl (K.K.); 3 Department of Chemistry, University of Life Sciences in Lublin, Akademicka 15, 20-950 Lublin, Poland; joanna.matysiak@up.lublin.pl; 4 Department of Cell Biology, Maria Curie-Sklodowska University, Akademicka 19, 20-033 Lublin, Poland

**Keywords:** agrochemicals, biotrophic fungi, heterocyclic compounds, plant diseases

## Abstract

Climate change forces agriculture to face the rapidly growing virulence of biotrophic fungal pathogens, which in turn drives researchers to seek new ways of combatting or limiting the spread of diseases caused by the same. While the use of agrochemicals may be the most efficient strategy in this context, it is important to ensure that such chemicals are safe for the natural environment. Heterocyclic compounds have enormous biological potential. A series of heterocyclic scaffolds (1,3,4-thiadiazole, 1,3-thiazole, 1,2,4-triazole, benzothiazine, benzothiadiazine, and quinazoline) containing 2,4-dihydroxylaryl substituents were investigated for their ability to inhibit the growth and development of biotrophic fungal pathogens associated with several important cereal diseases. Of the 33 analysed compounds, 3 were identified as having high inhibitory potential against *Blumeria* and *Puccinia* fungi. The conducted research indicated that the analysed compounds can be used to reduce the incidence of fungal diseases in cereals; however, further thorough research is required to investigate their effects on plant–pathogen systems, including molecular studies to determine the exact mechanism of their activity.

## 1. Introduction

Fungal leaf diseases are a major cause of economic losses in cereal cultivation. They reduce crop yields by hindering photosynthesis. Severe fungal infections can lead to defoliation and plant death. These diseases can also impact plant growth by altering nitrogen dynamics and the accumulation of carbohydrates in the grain [1,2,3,4]. Plant diseases emerge when there is a susceptible host, a virulent pathogen, and favourable environmental conditions [5,6,7], with the latter playing a crucial role in their development. Environmental changes can be closely linked to disease severity and associated losses [8,9]. The most economically significant and prevalent fungal diseases affecting cereals are powdery mildew and various types of rust.

The powdery mildew affecting cereals and grasses is a disease caused by the *Blumeria graminis* fungus. It affects all cereal species and many grass species. Its characteristic symptom is a white, powdery coating developing on the leaves, stems, and even ears [10,11].

The “rust fungi” (*Pucciniales*) are a diverse group of parasitic leaf pathogens that can cause significant economic and ecological harm. They reduce crop yields by forming rusty brown/orange powdery spores on leaf surfaces, which diminishes the plants’ ability to photosynthesise by redirecting photosynthesis to the fungi’s growth [12,13,14].

The rapid adaptation of plant pathogen populations to changing environmental conditions drives us to look for new, more effective solutions capable of protecting plants against fungal attacks. One effective approach is to enhance plants’ genetic resistance, but this process can take a long time. Alternatively, new chemicals can be developed to replace existing therapeutic strategies. Finding new molecules with fungicidal properties is currently one of the main priorities in organic chemistry, as the agrochemical industry is always on the search for new methods of effectively controlling fungal growth [15,16,17].

Heterocyclic compounds are a class of organic chemical compounds characterised by the presence of at least one atom of an element other than carbon in their ring structures [18]. They have long been under considerable scientific scrutiny due to their biological activity and synthetic utility [19,20]. The compounds exhibit various biological properties, including antifungal activity [21,22]. It is noteworthy that the antifungal activity of heterocyclic compounds depends, among other factors, on their structural composition. In particular, it has been reported that compounds comprising a 2,4-dihydroxyphenyl substituent show activity against various phytopathogens [23,24,25]. However, there are no reports in the available scientific literature regarding the activity of this type of compounds against biotrophic pathogens. Therefore, the aim of the presented work was to investigate the potential capacity of a diverse group of heterocyclic compounds containing a 2,4-dihydroxyphenyl substituent to inhibit the growth and development of biotrophic fungi causing the most common cereal diseases.

## 2. Results

Detailed tests results are presented in Table 1.

The initial test conducted at a concentration of 10 µg/mL yielded very promising results. Out of the 33 compounds analysed, 23 demonstrated an 80–100% capacity to actively inhibit the growth and development of *B. graminis* f.sp. *avenae*. Additionally, 25 compounds inhibited the growth and development of *B. graminis* f.sp. *tritici* at 50–100% efficiency, while 23 compounds were 80–100% effective in controlling the disease caused by *B. graminis* f.sp. *triticina*. Moreover, 22 of the analysed compounds inhibited the growth and development of all pathogens of the *Blumeria* genus, which are responsible for the incidence of powdery mildew in cereals and grasses.

Against *P. coronata* f.sp. *avenae*, development was inhibited with 80–100% efficiency by 17 compounds, while 18 compounds inhibited the development of *P. recondita* f.sp. *tritici* at 80–100%, and 20 compounds showed activity in inhibiting the growth of *P. hordei* by 50–100%. Furthermore, 17 compounds inhibited diseases caused by biotrophic fungi of the *Puccinia* genus in all of the analysed cereal species, and 16 were observed to effectively inhibit cereal diseases caused by pathogens from the *Blumeria* and *Puccinia* genera. All of these compounds reduced the incidence of disease by 80–100%. 

Further studies at lower concentrations revealed that some of the compounds could inhibit the growth and development of biotrophic fungal pathogens even at a concentration of 9 µg/mL, while a few were effective in controlling disease symptoms even at lower doses. The most promising results were recorded for compounds **28** and **29**, both of which inhibited the growth and development of all the analysed biotrophic fungi even at concentrations as low as 5 µg/mL. The compounds showed activity ranging from 50% against *Blumeria* fungi to 80–90% against pathogens causing crown rust in oats and brown rust in wheat. Compound **19** also demonstrated a high capacity to inhibit the growth and development of biotrophic fungi from the *Blumeria* and *Puccinia* genera, with 50% inhibition of *Blumeria* pathogens at a concentration of 6 µg/ mL and 50–80% inhibition of *Puccinia* pathogens at a concentration of 5 µg/mL.

A significant antifungal effect was also observed for methylsulfonylbenzothiadiazine (compound **27**), in particular against *B. graminis* f.sp. *avenae* and *P. coronata* f.sp. *avenae*, benzothiazinethione (compound **21**) against *P. hordei,* and thiazolopyrimidinone (compound **21**) against *B. graminis* f.sp. *avenae*. 

## 3. Discussion

The primary challenge faced by modern agriculture is the need to ensure the availability of sufficient high-quality food to meet the needs of a constantly growing population. The production of necessary food, feed, fuel, and fibre increasingly requires innovative solutions to challenges posed by climate change and diseases mediated by pathogenic fungi [26,27].

From the perspective of integrated plant protection, the most effective method of reducing the incidence of diseases is the development and cultivation of genetically resistant varieties [28]. However, the interaction between plants and pathogens is dynamic and often unpredictable [27]. Through mutation and genetic recombination, pathogens can develop new virulence factors and attack previously resistant varieties [29]. Moreover, climatic conditions favourable to the development of pathogens may contribute to the emergence of new, virulent pathotypes [30,31,32].

In such cases, genetic resistance does not always fully protect the plant against fungal infection. When genetic resistance proves incomplete, fungicides can be used to complement the weakened resistance of the host plant. Foliar fungicides are the final line of defence in the integrated disease management (IDM) approach to controlling cereal diseases. These chemicals are used to compensate for a given variety’s susceptibilities [33,34]. Analyses conducted in major crops, such as rice, wheat, barley, and corn, indicate that without additional plant protection, yields would decrease by about 50% [26].

While modern agricultural chemicals are able to address a variety of farming challenges, the side effects of agrochemicals on crop yield and quality are an increasingly significant factor. Agrochemical companies that prioritise research and development have the potential to influence the future of agriculture by offering innovative, comprehensive solutions. To ensure high efficiency and quality in food, feed, fibre, and fuel production, sustainable agricultural practices that are economically, ecologically, and environmentally sound are essential [7,26,35].

It is, therefore, crucial to explore new chemical compounds that can inhibit the growth and development of fungal pathogens without being harmful to the environment and living organisms [36,37]. Numerous scientific reports point to heterocyclic compounds as promising solutions for agriculture [26,36]. In our study, we aimed to determine whether different agents from this group can provide viable alternatives effective against biotrophic fungal pathogens in cereals. The research focused on compounds with diverse structures, all of which featured heterocyclic components. All of the compounds featured a 2,4-dihydroxy moiety, in some cases modified with a Cl, Me, Et, or OH substituent. This specific structural composition is responsible for the hydrophilic–lipophilic character of the agents as well as their solubility in water. Moreover, hydroxyl groups may participate in the interactions with potential molecular targets (hydrogen bond donors and acceptors).

Heterocyclic compounds are currently under considerable research scrutiny due to their potential applicability as plant protection agents and in the treatment of plant diseases caused by fungi. The literature confirms that derivatives of compounds belonging to the analysed groups show antimycotic properties, including against fungi attacking plants [38,39]. Many laboratories are currently hard at work testing the effectiveness of heterocyclic compounds against plant pathogens. Notably, the properties of these compounds are not limited only to antifungal effects, but also include bactericidal, anti-inflammatory, anticancer, and antiviral activity [40]. A number of studies have demonstrated that compounds containing the resorcinol-heterocyclic ring, including 5-modified 4-(1,3,4-thiadiazol-2-yl)benzene-1,3-diols and the N-substituted 2,4-dihydroxybenzenecarbothioamides moiety are effective against phytopathogenic fungi [23,41]. The most active of the studied agents exhibited fungistatic effects with 90–100% efficiency when concentrated at 20 µg/mL. The antifungal potential of some compounds has also been confirmed in in vivo conditions [23]. 

The present research confirmed promising biological activity against biotrophic fungal pathogens in a significant number of the tested compounds exhibited, with the strongest effects observed in certain derivatives of quinazoline (compounds **28** and **29**) and triazolopyridazine (compound **19**). 

Quinazolines and their derivatives are among the most active agents in this group, showing a wide range of biological activities, including antibacterial, analgesic, antimicrobial, anti-inflammatory, anticancer, antihypertensive, antifungal, anti-HIV, inhibitory, analgesic, medicinal, antiprotozoal, antitumor, and anti-tubercular properties [42,43,44]. Quinazolines and their derivatives have also been used as disease control agents in crops. In our study, the quinazoline derivative showed high effectiveness in inhibiting the growth and development of biotrophic fungi that are attacking the most common cereal species.

Pyridazine is a common heterocyclic core containing nitrogen and found in a wide variety of molecules with diverse biological characteristics [45,46]. Triazoles are commonly employed in medicinal chemistry [47,48]. Mohgimi et al. (2021) [49] demonstrated the strong biological potential of a pyridazine triazole hybrid as an α-glucosidase inhibitor. Our own studies confirmed that compounds containing a triazolopyridazine fused ring are particularly efficient in inhibiting the growth and development of biotrophic fungal pathogens.

## 4. Materials and Methods

The study was conducted on 33 compounds obtained at the Department of Chemistry of the University of Life Sciences in Lublin. They were synthesised through endocyclisation of aryl-modified (or not) sulphinylbis(2,4-dihydroxyphenyl)methanethiones with corresponding nucleophiles [50,51,52,53,54]. The compounds selected for the analyses were divided into the following groups: 

1,3,4-thiadiazole derivatives: 5-(2,4-dihydroxyphenyl)-1,3,4-thiadiazole-2-carboxamide (1), 4-(5-(2,5-bis(2,2,2-trifluoroethoxy)phenyl)-1,3,4-thiadiazol-2-yl)-6-ethylbenzene-1,3-diol (2), 4-(5-(naphthalen-1-ylamino)-1,3,4-thiadiazol-2-yl)benzene-1,3-diol (3), 4-(5-(1-(4-chlorophenyl)-5-phenyl-1*H*-pyrazol-4-yl)-1,3,4-thiadiazol-2-yl)-6-ethylbenzene-1,3-diol (4), 4-ethyl-6-(5-(6-morpholinopyridin-3-yl)-1,3,4-thiadiazol-2-yl)benzene-1,3-diol (5), 4-(5-(5-bromo-1-methyl-1*H*-indol-2-yl)-1,3,4-thiadiazol-2-yl)-6-ethylbenzene-1,3-diol (6), 4-(5-(2-methylimidazo[1,2-a]pyridin-3-yl)-1,3,4-thiadiazol-2-yl)benzene-1,3-diol (7), 4-(5-(3-bromoimidazo[1,2-a]pyridin-7-yl)-1,3,4-thiadiazol-2-yl)benzene-1,3-diol (8), 4-(5-(benzo[*c*][1,2,5]oxadiazol-5-yl)-1,3,4-thiadiazol-2-yl)-6-ethylbenzene-1,3-diol (9), 4-(5-((2-(trifluoromethyl)quinolin-4-ylthio)methyl)-1,3,4-thiadiazol-2-yl)benzene-1,3-diol (10), 5-(2,4-dihydroxyphenyl)-N-(2,4-dioxo-1,2,3,4-tetrahydropyrimidin-5-yl)-1,3,4-thiadiazole-2-carbothioamide (11); 1,3-tiazole fused derivatives (thiazolopyrimidines and naphthothiazole): 7-amino-2-(2,4-dihydroxyphenyl)-3a-hydroxy-4,6-dimethyl-3*a*,4-dihydrothiazolo[5,4-*d*]pyrimidin-5(6*H*)-one (12), 2-(2,4-dihydroxyphenyl)-3*a*-hydroxy-3*a*,4-dihydrothiazolo[5,4-*d*]pyrimidin-5(6*H*)-one (13) 2-(5-chloro-2,4-dihydroxyphenyl)-3a-hydroxy-3a,4-dihydrothiazolo[5,4-*d*]pyrimidin-5(6*H*)-one (14), 2-(2,4-dihydroxy-3-methylphenyl)naphtho[2,3-*d*]thiazole-4,9-dione (15); 1,2,4--triazole fused derivatives: 4-(6-bromo-[1,2,4]triazolo[4,3-*a*]pyridin-3-yl)-6-chlorobenzene-1,3-diol (16), 4-(5-(4-methoxyphenyl)-7-(trifluoromethyl)-[1,2,4]triazolo[4,3-*a*]pyrimidin-3-yl)benzene-1,3-diol (17), 4-([1,2,4]triazolo [3,4-*a*]phthalazin-3-yl)benzene-1,3-diol (18), 4-(5-(6-chloro-[1,2,4]triazolo[1,5-*b*]pyridazin-2-yl)-1,3,4-thiadiazol-2-yl)benzene-1,3-diol (19); 4-(3-phenylthiazolo[2,3-*c*][1,2,4]triazol-6-yl)benzene-1,3-diol (20);

Benzotiazine derivatives: 2-(2,4-dihydroxyphenyl)-4*H*-benzo[*d*][1,3]thiazine-4-thione (21), 2-(2,3,4-trihydroxyphenyl)-3′*H*-spiro[benzo[*d*][1,3]thiazine-4,1′-isobenzofuran]-3′-one (22), 2-(2,4-dihydroxyphenyl)-3*a*-hydroxy-3*aH*-[1,3]thiazino[6,5,4-*cd*]isoindol-5(4*H*)-one (23);

Benzothiadiazine derivatives: 4-(6,8-dichloro-1*H*-benzo[e][1,3,4]thiadiazin-3-yl)benzene-1,3-diol (24), 4-(5,7,8-trifluoro-1*H*-benzo[*e*][1,3,4]thiadiazin-3-yl)benzene-1,3-diol (25), trifluoromethyl 3-(2,4-dihydroxyphenyl)-1*H*-benzo[*e*][1,3,4]thiadiazine-6-sulfonate (26), 2-methyl-4-(7-methyl-6-(methylsulfonyl)-1*H*-benzo[*e*][1,3,4]thiadiazin-3-yl)benzene-1,3-diol (27);

Quinazoline derivatives: 4-chloro-6-(5-fluoro-3,4-dihydroquinazolin-2-yl)benzene-1,3-diol (28), 4-ethyl-6-(7-(trifluoromethyl)-3,4-dihydroquinazolin-2-yl)benzene-1,3-diol (29) and the other derivatives: 4-chloro-6-(1-phenyl-1*H*-benzo[*d*]imidazol-2-yl)benzene-1,3-diol (30), 2-(2,4-dihydroxy-3-methylphenyl)-4*H*-benzofuro[3,2-*d*][1,3]thiazin-4-one (31), 6-(2,4-dihydroxyphenyl)-1-phenylpyrazolo[3,4-*d*][1,3]thiazin-4(1*H*)-one (32, 33).

The structures of the compounds are shown in Figure S1.

The antifungal activity of the compounds was tested in vitro against the most common biotrophic fungal pathogens infecting cereals (Table 2).

The target compounds were dissolved in DMSO to obtain a stock solution concentrated at 100 μg/mL. In the first test, 100 µL of the compound was added to 10 mL of agar medium (6 g/L) to obtain a concentration of 10 μg/mL. In further experiments aimed at determining the minimum amount of the compound capable of inhibiting the growth and development of fungi, the tested concentrations of the compounds were, respectively, 9, 8, 7, 6, and 5 μg/mL. Leaves obtained from cultivars susceptible to the analysed pathogens (Fuchs (*A. sativa*), Błyskawica (*T. aestivum*), Klaus (x *Triticosecale*), and Mecenas (*H. vulgare*)) were placed on Petri dishes containing the agar medium with the addition of the analysed compounds. Control leaves were placed into petri plates containing agar supplemented with DMSO. Petri dishes with leaf fragments were inoculated with pathogen spores in accordance with the host–pathogen test methodology described by [55]. The plates were then incubated in a growing chamber at 17 °C and under an illuminance of approximately 4 kLx. After 10 days, the extent of leaf infection was assessed using the 5-point scale presented in Table 3. 

The percentage results representing the effectiveness of disease control were calculated relative to the control plants, with 100% indicating complete disease control and 0% indicating the absence of disease control [57]. 

## 5. Conclusions

Our research demonstrated that numerous representatives of the diverse group of heterocyclic compounds containing a 2,4-dihydroxyphenyl substituent have the capacity to inhibit the growth of biotrophic fungal pathogens in cereals. Moreover, we were able to identify compounds with a broad spectrum of relevant inhibitory activity, particularly against *Blumeria* and *Puccinia* pathogens that are attacking some of the most important cereal species. The results indicate the possibility of developing new, advanced fungicides with potential agricultural applications. Moreover, the proven non-toxicity of these compounds renders them even more viable in sustainable farming, underscoring the need for further study on the mechanisms of their activity and interaction within the plant–pathogen system.

## Figures and Tables

**Table 1:** Percentage disease control by tested compounds. 100 indicates complete disease control, while 0 indicates a lack of disease control.

Compound Groups	Compound	Fungal Pathogens																																			
		*Blumeria graminis* f.sp. *avenae*,						*Blumeria graminis* f.sp. *tritici*						*B. graminis* f. sp. *triticale*						*Puccinia coronata* f.sp. *avenae*						*Puccinia recondita* f.sp. *tritici*.						*Puccinia hordei*					
		Compound Concentration μg/mL																																			
		10	9	8	7	6	5	10	9	8	7	6	5	10	9	8	7	6	5	10	9	8	7	6	5	10	9	8	7	6	5	10	9	8	7	6	5
Control	DMSO	0	0	0	0	0	0	0	0	0	0	0	0	0	0	0	0	0	0	0	0	0	0	0	0	0	0	0	0	0	0	0	0	0	0	0	0
1,3,4-thiadiazole derivatives	1	0	0	0	0	0	0	0	0	0	0	0	0	0	0	0	0	0	0	0	0	0	0	0	0	0	0	0	0	0	0	50	0	0	0	0	0
	2	90	0	0	0	0	0	80	0	0	0	0	0	100	0	0	0	0	0	100	90	0	0	0	0	80	0	0	0	0	0	80	0	0	0	0	0
	3	0	0	0	0	0	0	0	0	0	0	0	0	0	0	0	0	0	0	0	0	0	0	0	0	0	0	0	0	0	0	0	0	0	0	0	0
	4	90	0	0	0	0	0	90	0	0	0	0	0	80	0	0	0	0	0	0	0	0	0	0	0	0	0	0	0	0	0	0	0	0	0	0	0
	5	100	0	0	0	0	0	100	0	0	0	0	0	100	0	0	0	0	0	90	0	0	0	0	0	90	0	0	0	0	0	100	0	0	0	0	0
	6	80	0	0	0	0	0	100	0	0	0	0	0	100	0	0	0	0	0	0	0	0	0	0	0	0	0	0	0	0	0	0	0	0	0	0	0
	7	100	0	0	0	0	0	100	0	0	0	0	0	100	0	0	0	0	0	100	0	0	0	0	0	100	0	0	0	0	0	100	0	0	0	0	0
	8	100	0	0	0	0	0	100	0	0	0	0	0	100	0	0	0	0	0	90	0	0	0	0	0	90	0	0	0	0	0	100	0	0	0	0	0
	9	100	0	0	0	0	0	90	0	0	0	0	0	80	0	0	0	0	0	0	0	0	0	0	0	0	0	0	0	0	0	0	0	0	0	0	0
	10	0	0	0	0	0	0	0	0	0	0	0	0	0	0	0	0	0	0	0	0	0	0	0	0	0	0	0	0	0	0	0	0	0	0	0	0
	11	80	0	0	0	0	0	100	0	0	0	0	0	100	0	0	0	0	0	0	0	0	0	0	0	80	0	0	0	0	0	100	90	0	0	0	0
1,3-tiazole fused derivatives	12	100	0	0	0	0	0	100	0	0	0	0	0	100	0	0	0	0	0	100	0	0	0	0	0	100	0	0	0	0	0	100	0	0	0	0	0
	13	100	80	50	0	0	0	50	0	0	0	0	0	100	0	0	0	0	0	100	90	0	0	0	0	100	90	0	0	0	0	90	0	0	0	0	0
	14	90	0	0	0	0	0	90	0	0	0	0	0	50	0	0	0	0	0	80	0	0	0	0	0	80	0	0	0	0	0	80	0	0	0	0	0
	15	0	0	0	0	0	0	0	0	0	0	0	0	0	0	0	0	0	0	0	0	0	0	0	0	0	0	0	0	0	0	0	0	0	0	0	0
1,2,4-triazole fused derivatives	16	100	0	0	0	0	0	100	0	0	0	0	0	100	0	0	0	0	0	100	80	0	0	0	0	100	0	0	0	0	0	100	0	0	0	0	0
	17	0	0	0	0	0	0	0	0	0	0	0	0	0	0	0	0	0	0	0	0	0	0	0	0	0	0	0	0	0	0	0	0	0	0	0	0
	18	90	0	0	0	0	0	90	0	0	0	0	0	0	0	0	0	0	0	80	0	0	0	0	0	80	0	0	0	0	0	100	0	0	0	0	0
	19	100	90	80	50	50	0	100	90	80	50	50	0	100	90	80	50	50	0	100	100	100	100	80	80	100	100	100	90	80	80	100	90	80	70	50	50
	20	100	0	0	0	0	0	100	0	0	0	0	0	100	0	0	0	0	0	100	90	0	0	0	0	100	0	0	0	0	0	100	0	0	0	0	0
Benzothiazine derivatives	21	100	0	0	0	0	0	100	0	0	0	0	0	100	0	0	0	0	0	100	50	0	0	0	0	80	50	0	0	0	0	100	80	50	0	0	0
	22	90	0	0	0	0	0	100	0	0	0	0	0	100	0	0	0	0	0	100	0	0	0	0	0	100	0	0	0	0	0	90	80	0	0	0	0
	23	0	0	0	0	0	0	0	0	0	0	0	0	0	0	0	0	0	0	0	0	0	0	0	0	0	0	0	0	0	0	0	0	0	0	0	0
Benzothiadiazine derivatives	24	0	0	0	0	0	0	50	0	0	0	0	0	0	0	0	0	0	0	0	0	0	0	0	0	0	0	0	0	0	0	0	0	0	0	0	0
	25	80	0	0	0	0	0	80	0	0	0	0	0	80	0	0	0	0	0	0	0	0	0	0	0	0	0	0	0	0	0	0	0	0	0	0	0
	26	0	0	0	0	0	0	0	0	0	0	0	0	0	0	0	0	0	0	0	0	0	0	0	0	0	0	0	0	0	0	0	0	0	0	0	0
	27	100	90	80	50	50	0	90	0	0	0	0	0	90	0	0	0	0	0	100	100	90	90	80	0	100	100	0	0	0	0	90	0	0	0	0	0
Quinazoline derivatives	28	100	90	80	80	50	50	100	90	90	80	50	50	100	90	80	80	50	50	100	100	100	90	90	90	100	100	100	90	90	80	100	90	80	70	50	50
	29	100	100	80	80	50	50	100	80	80	80	50	50	100	80	80	50	50	50	100	100	100	90	90	90	100	100	100	90	90	80	100	80	70	50	50	50
	30	80	0	0	0	0	0	100	0	0	0	0	0	100	0	0	0	0	0	0	0	0	0	0	0	0	0	0	0	0	0	0	0	0	0	0	0
	31	0	0	0	0	0	0	0	0	0	0	0	0	0	0	0	0	0	0	0	0	0	0	0	0	0	0	0	0	0	0	0	0	0	0	0	0
	32	0	0	0	0	0	0	100	80	0	0	0	0	100	50	0	0	0	0	0	0	0	0	0	0	0	0	0	0	0	0	90	0	0	0	0	0
	33	90	0	0	0	0	0	90	0	0	0	0	0	90	0	0	0	0	0	100	80	80	0	0	0	100	80	0	0	0	0	100	80	0	0	0	0

**Table 2:** Pathogens and hosts used in the study.

Cereal	Pathogen	Disease
Oat	*Blumeria graminis* (DC.) Speer f.sp. *avenae* Marchal	Powdery mildew
	*Puccinia coronata* Corda f.sp. *avenae*	Crown rust
Wheat	*Blumeria graminis* (DC.) Speer f.sp. *tritici* Marchal	Powdery mildew
	*Puccinia recondita* Rob. ex Desm f.sp. *tritici* (Eriks.)	Brown rust
Barley	*Puccinia hordei* Otth.	Leaf rust
Triticale	*B. graminis* (DC.) Speerf. sp. *triticale*	Powdery mildew

**Table 3:** The 5-point scale used to assess the extent of fungal infection [56].

Degree of Infection	Description of the Degree	Effectiveness of Disease Control in %
0	No symptoms	100%
1	Limited development of the pathogen in small, singular colonies	90%
2	Mycelium visible with a small quantity of spores—less than 20% of the leaf surface	80%
3	Extensive mycelium occupying 20–50% of the leaf surface	50%
4	Abundant mycelium occupying more than 50% of the leaf surface	0%

## Data Availability

Data are available upon request from the corresponding authors.

## Supplementary Materials

The following supplementary materials can be downloaded at https://www.mdpi.com/article/10.3390/ijms25158262/s1.
