# Supplementary figures and images for: Investigation of 2,4-Dihydroxylaryl-Substituted Heterocycles as Inhibitors of the Growth and Development of Biotrophic Fungal Pathogens Associated with the Most Common Cereal Diseases
Source: Int J Mol Sci. 2024 Jul 29;25(15):8262. doi: 10.3390/ijms25158262 (PMC11312687; doi:10.3390/ijms25158262)

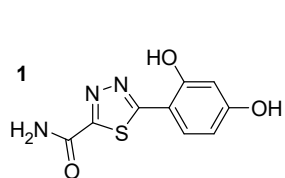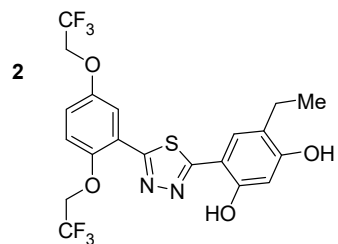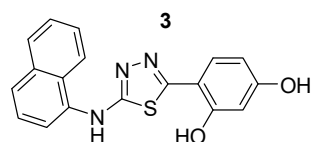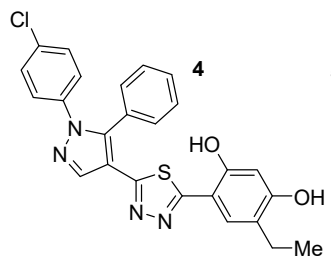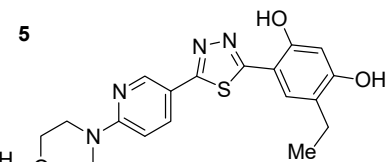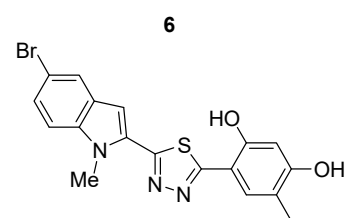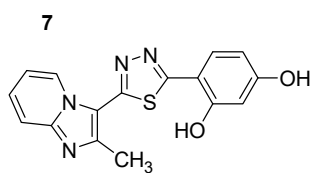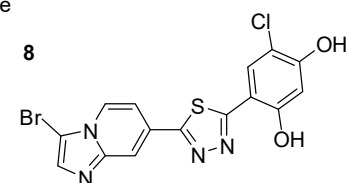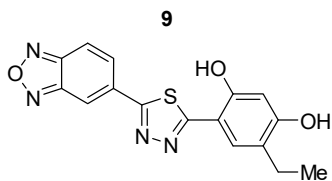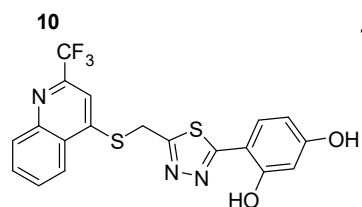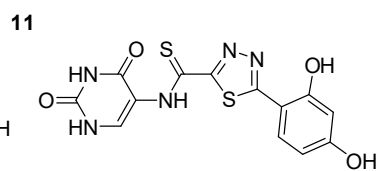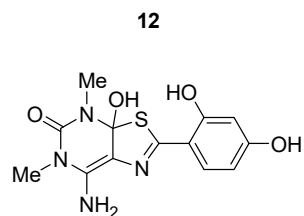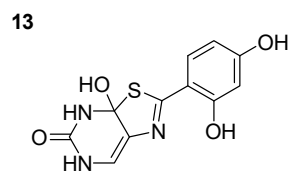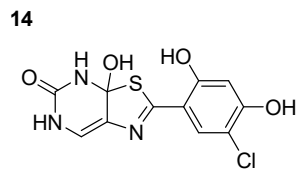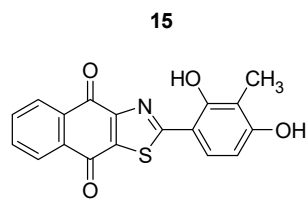

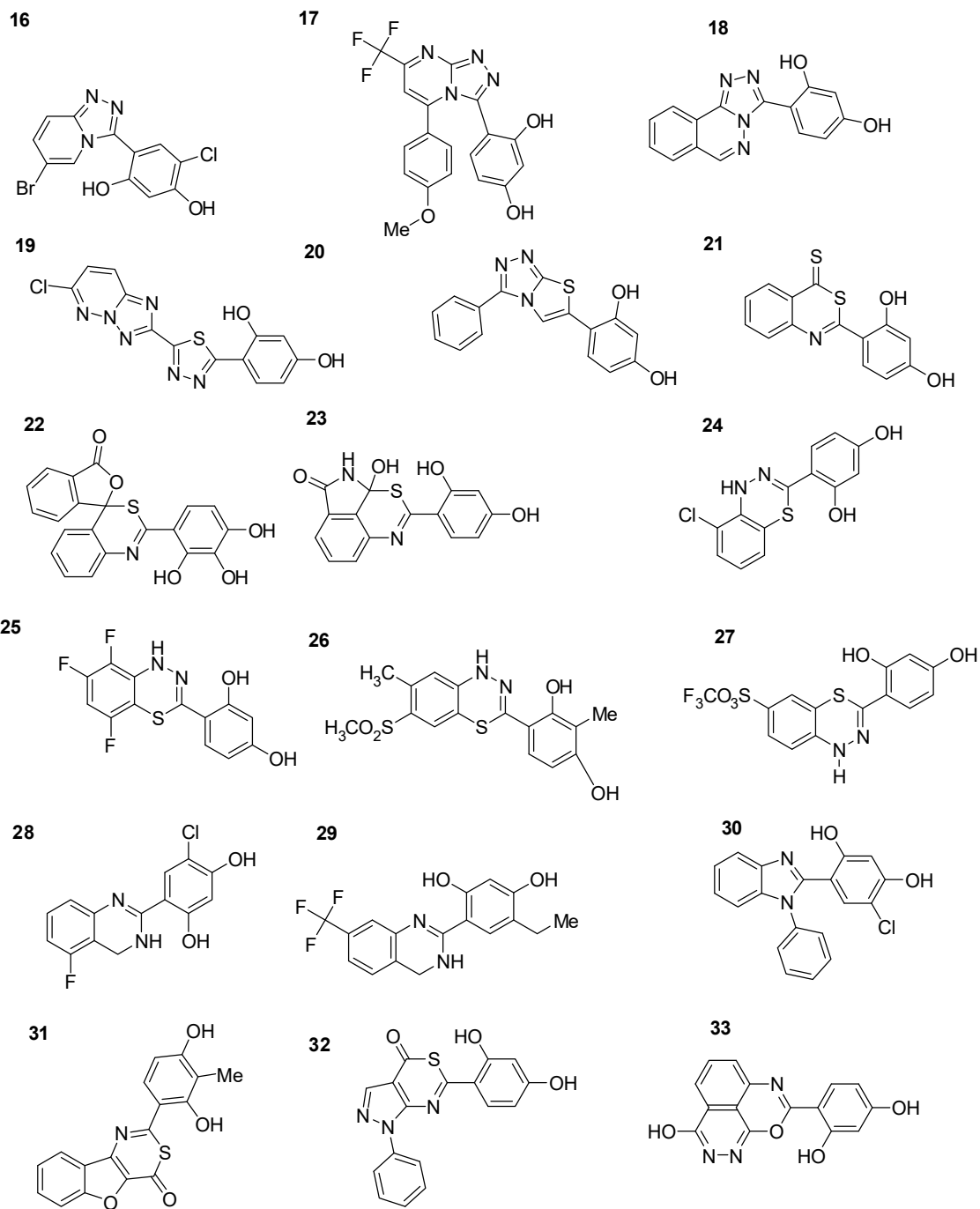

Figure S1. The structure of analyzed compounds 1-33

Supplement: Supplementary file 1 [file ijms-25-08262-s001.zip › ijms-3092377-supplementary.pdf]
